# Supplementary material for: Enhanced Stability of Vegetal Diamine Oxidase with Trehalose and Sucrose as Cryoprotectants: Mechanistic Insights
Source: Molecules. 2023 Jan 19;28(3):992. doi: 10.3390/molecules28030992 (PMC9921882; doi:10.3390/molecules28030992)
Supplement: Supplementary file 1 [file molecules-28-00992-s001.zip › molecules-2120925-supplementary.pdf]

molecules

MDPI

Supplementary Materials

Enhanced Stability of Vegetal Diamine Oxidase with Trehalose and Sucrose as Cryoprotectants: Mechanistic Insights

Meriem Megoura, Pompilia Ispas-Szabo and Mircea Alexandru Mateescu *

Research Chair on Enteric Dysfunctions "Allerdys" & CERMO-FC Center, Department of Chemistry, Faculty of Science, Université du Québec à Montréal, C.P. 8888, Branch Centre-Ville, Montréal (QC) H3C 3P8, Canada; megoura.meriem@courrier.uqam.ca (M.M.); ispas-szabo.pompilia@uqam.ca (P.I.-S.)

* Correspondence: mateescu.m-alexandru@uqam.ca (M.A.M); Tel.: +1-514-987-4319

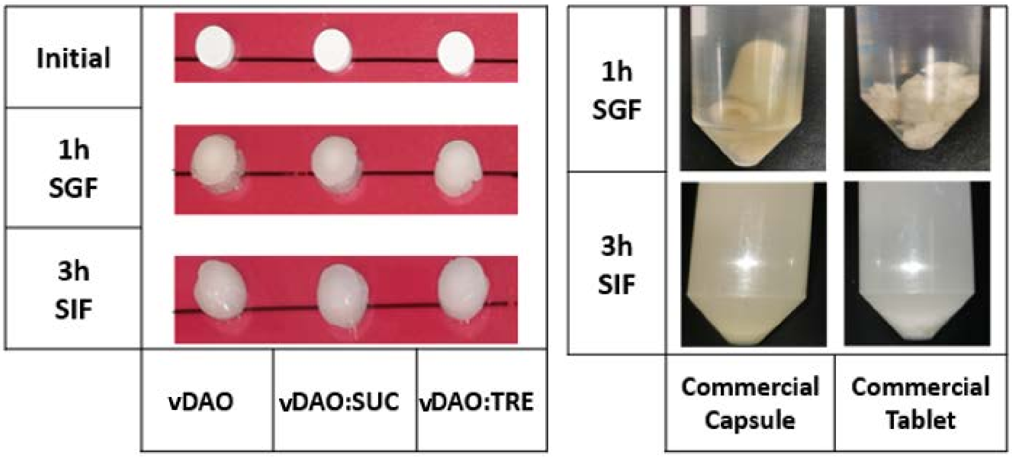

Figure S1. Comparison of behavior for tablets obtained with our preparations: vDAO (vegetal diamine oxidase), vDAO:SUC (vDAO formulated either with sucrose), and vDAO:TRE (vDAO formulated either with trehalose) (**Left**) versus commercial tablet and capsule (**Right**) all tested in the same conditions in simulated gastric and intestinal fluids.

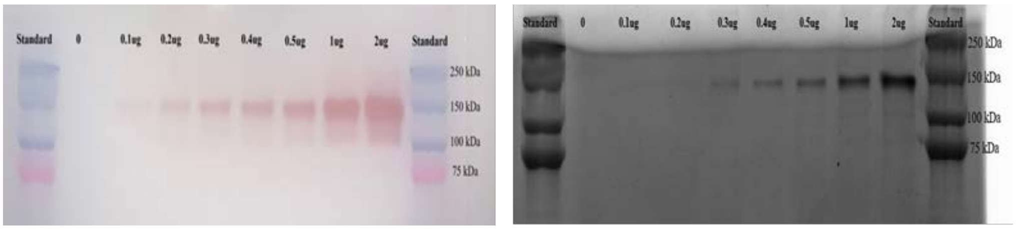

Figure S2. Detection of vDAO activity with the DCHBS-AAP method. Zymography of vDAO (0-2 µg/well) developed with DCHBS-AAP (**Left**) for vDAO activity and restained using Coomassie Brilliant Blue (**Right**) on SDS-Polyacrylamide (10%) gel electrophoresis (representative of *n* = 3 runs).
